# A 46-year-old woman with a history of personality change, headache, and olfactory hallucination

**Published:** 2013

**Authors:** Ali Okhovat

**Affiliations:** Neurologist, Sina Hospital, Tehran University of Medical Sciences, Tehran, Iran

**Keywords:** Olfactory Hallucination, Meningioma, Headache, Suprasellar

## Case

The patient was a 46-year-old woman, who was visited in our hospital because of progressive personality change, visual field defect, severe headache, and olfactory hallucination for the previous 3 months.

On neurological examination, she had hyposmia and bitemporal hemianopia. Mini-mental status examination score was 28 out of 30. Furthermore, he had bilateral papilledema. Other neurological examination was normal.

**Figure 1:**
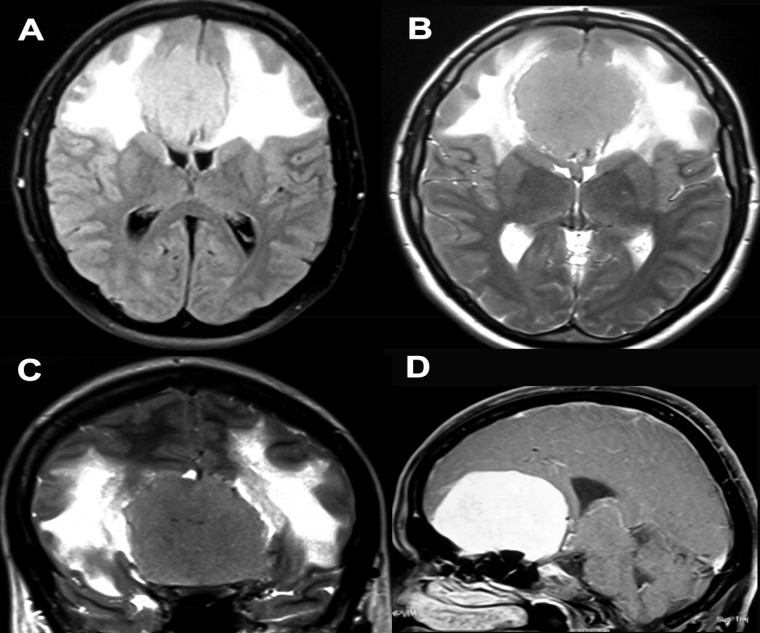
Brain magnetic resonance imaging (MRI) of the patient: Axial FLAIR and T2-weighted brain MRI with hypersignal lesion in midline of frontal area (A, B), coronal FLAIR demonstrating the mass with intense edema (C), and T1-weighted sagittal MRI indicating avidly enhancement of the mass after contrast administration

The brain magnetic resonance imaging (MRI) is presented in Figure 1:

1. What abnormalities are seen on brain MRI?
2. What are the best differential diagnoses?
3. What is the most probable diagnosis?
4. What is the best treatment for this patient?
5. What are the possible complications of treatment?
6. What are the differential diagnoses of suprasellar mass?

## Answers

1. The brain MRI shows anterior midline large extra-axial mass with massive surrounding edema and intense enhancement with gadolinium.
2. The differential diagnosis is usually limited, and the MRI findings are usually very characteristic of these tumors. An olfactory groove meningioma and olfactory nerve schwannoma or subfrontal schwannoma not associated with any cranial nerves can arise in that location.
3. Olfactory groove meningioma
4. The treatment of olfactory groove meningioma usually requires surgery at the time of diagnosis.
5. The most common reported complication is the presence of a postoperative cerebrospinal fluid (CSF) leak; other complications include infections, visual deterioration, and ischemic strokes.

The differential diagnoses of suprasellar mass include neoplastic, vascular, congenital, or infectious/inflammatory suprasellar masses. In detail, they consist of tumors [chiasmatic astrocytoma, chordoma, craniopharyngioma, dermoid (CNS)/epidermoid/intracranial teratoma, germinoma, granular cell tumor of the pituitary, hypothalamic astrocytoma/glioma, meningioma, optic nerve glioma, pilocytic astrocytoma of the neurohypophysis (infundibuloma), pituitary adenoma (the most common in the adult population), and pituitary metastases], cellular infiltrates (Langerhans cell histiocytosis, lymphocytic hypophysitis, sarcoidosis/sarcoid granuloma), and other lesions [anterior circulation berry aneurysm, hamartoma (tuber cinereum hamartoma), intracranial lipoma, pituitary abscess, pituitary stone, Rathke's cleft cyst, and sphenoid sinus mucocele].

## Discussion

Olfactory groove meningiomas (OGM) arise from the midline of the anterior fossa at ethmoidal cribriform plate and account around 10% of all intracranial meningiomas.^1^ OGM can progress towards the sella region and if large enough, it can affect vision by compressing the optic nerve and chiasm.^2^

In one study, the most frequent clinical finding was headache (87%), followed by anosmia (78%), personality changes (63%), visual impairment (61%), increased intracranial pressure (ICP) syndrome (51%), and seizures (36%).^3^

The time from the development of initial symptoms to the time of diagnosis varies between different studies and has been reported to be up to 14 years.^1^ MRI with and without gadolinium is the imaging of choice to confirm the diagnosis of an OGM. The appearance of these tumors on MRI is similar to that of meningiomas found in other regions of the brain or spinal canal. OGM appears as a homogeneously intense enhancing lesion with a dural base located on the cribriform plate. OGMs are usually isointense to gray matter on T1-weighted sequences and iso- to hyperintense on T2-weighted sequences.^4^

OGMs usually require surgical treatment at the time of diagnosis because of their size and associated compression effect on surrounding brain tissue. Several surgical procedures are commonly used for treatment of OGMs (depending on the size, location, and the extension of the tumor) including the subfrontal, pterional, or inter-hemispheric approaches.^5, 6^

The most common reported surgery complication of OGMs is the postoperative CSF leak, which occurs in 7% of patients. Infections (meningitis) are more frequent after surgery of OGMs than after craniotomies for other indications. Visual deterioration is thought to be occurred secondary to ischemic damage to the optic nerve. Ischemic strokes are rare and are related to the dissection of the tumor capsule along neurovascular structures.^1, 3^

The recurrence rate depends on the degree of initial resection, length of follow-up, and the degree of orbital or paranasal sinus involvement (up to 40%). Recurrence rates for OGMs are higher than those of meningiomas affecting the cerebral convexities. The best treatment option of recurrence depends on the location and size. Surgery may be mandatory in cases of large tumors and meticulous resection of abnormal bone and paranasal disease should be undertaken. Radiosurgery may be useful to prevent the development of further recurrence.^7, 8^
